# Diffusion of Quinine with Ethanol as a Co-Solvent in Supercritical CO_2_

**DOI:** 10.3390/molecules25225372

**Published:** 2020-11-17

**Authors:** Yury Gaponenko, Aliaksandr Mialdun, Valentina Shevtsova

**Affiliations:** 1 MRC—Microgravity Research Centre, Université libre de Bruxelles (ULB), EP-CP165/62, Avenue F.D. Roosevelt 50, B-1050 Brussels, Belgium; ygaponen@ulb.ac.be (Y.G.); amialdun@ulb.ac.be (A.M.); 2 Mechanical and Manufacturing Department, Mondragon Goi Eskola Politeknikoa (MGEP), Loramendi 4, Apdo. 23, 20500 Mondragon, Spain; 3 IKERBASQUE, Basque Foundation for Science, 48009 Bilbao, Spain

**Keywords:** supercritical CO_2_, quinine, diffusion coefficients, ethanol cosolvent, Taylor dispersion

## Abstract

This study aims at contributing to quinine extraction using supercritical CO_2_ and ethanol as a co-solvent. The diffusion coefficients of quinine in supercritical CO_2_ are measured using the Taylor dispersion technique when quinine is pre-dissolved in ethanol. First, the diffusion coefficients of pure ethanol in the supercritical state of CO_2_ were investigated in order to get a basis for seeing a relative change in the diffusion coefficient with the addition of quinine. We report measurements of the diffusion coefficients of ethanol in scCO_2_ in the temperature range from 304.3 to 343 K and pressures of 9.5, 10 and 12 MPa. Next, the diffusion coefficients of different amounts of quinine dissolved in ethanol and injected into supercritical CO_2_ were measured in the same range of temperatures at *p* = 12 Mpa. At the pressure *p* = 9.5 MPa, which is close to the critical pressure, the diffusion coefficients were measured at the temperature, *T* = 343 K, far from the critical value. It was found that the diffusion coefficients are significantly dependent on the amount of quinine in a small range of its content, less than 0.1%. It is quite likely that this behavior is associated with a change in the spatial structure, that is, the formation of clusters or compounds, and a subsequent increase in the molecular weight of the diffusive substance.

## 1. Introduction

Extraction using supercritical carbon dioxide has got rapid development in the last few years. Supercritical CO_2_ is considered to be a green solvent due to the fact that it is not toxic, non-flammable, inert, easily processable and offers moderate critical temperature and pressure. In its gaseous form, CO_2_ is essentially a non-solvent but, in the supercritical fluid state above its critical point ($T_{cr}$ = 304.13 K and $p_{cr}$ = 7.38 MPa), the density and solvation capabilities of CO_2_ change dramatically. The key physico-chemical properties of a supercritical fluid such as density, diffusivity and dielectric constant can be easily controlled by changing the pressure and/or temperature. It is also a clean and versatile solvent compared to organic solvents and chlorofluorocarbons.

Extraction of different compounds from plants can be obtained by the number of processes such as mechanical pressing and grinding, maceration, solvent extraction and distillation. Supercritical fluid extraction with CO_2_ is a promising alternative due to the fact that it can easily be separated from the product due to adsorption, absorption or evaporation. This offers good product purity as none of the above separation processes can be detrimental to the product.

Quinine, being one of the alkaloids, is considered to be the superior antimalarial drug as it can be used to treat against the plasmodium that has proven to be resistant to other antimalarials. Quinine is not only an anti-malarial, it has also antipyretic, analgesic and anti-inflammatory properties which gives it the ability to treat arthritis, lupus and leg cramps [1]. It is also widely used in beverages such as tonic water. Traditional methods of quinine extraction have proven to be not only expensive but also difficult to get pure quinine without the presence of such contaminants as dyes, resins and other compounds present in the bark. Most of these extraction and preparation methods employ the use of strong acids or petrochemical-derived solvents (e.g., hexane), and as a result, quinine is obtained in the form of salts such as sulphates, bisulphates, chlorides, dihydrochlorides and gluconates [2]. The current research is focused on contribution to the development of an extraction method for quinine using supercritical CO_2_.

However, supercritical CO_2_ is not well suitable for the extraction of high molecular weight compounds like quinine. Ability of supercritical CO_2_ for the extraction of high molecular weight and polar compounds can be improved by adding a polar modifier as a co-solvent. For this, a modifier needs to have a high affinity for supercritical CO_2_ in targeted solute (quinine, in this case), increase the solubility of the solute and be generally safe for use [3,4]. Ethanol is a good candidate for the use as a modifier, as it worked well with supercritical CO_2_ to extract a number of carotenoids from plant materials, such as carrot roots, red pepper, tomato fruit, and apricot fruit [4]. A good example of the use of ethanol as a modifier in industry is the extraction of essential oils from plants and herbs using supercritical CO_2_ that contains up to 10% of ethanol [3].

## 2. Experimental

### 2.1. Materials

CO_2_ with a certified purity of 0.99998 mol mol${}^{-1}$ was purchased from Air Liquide in a bottle in its vapor-liquid equilibrium state, i.e., with a nominal pressure *p* = 6.4 MPa at *T* = 298 K. Ethanol was purchased from VMR in a purity of 99.9% in volume fraction and used without further purification.

**Figure 1:**
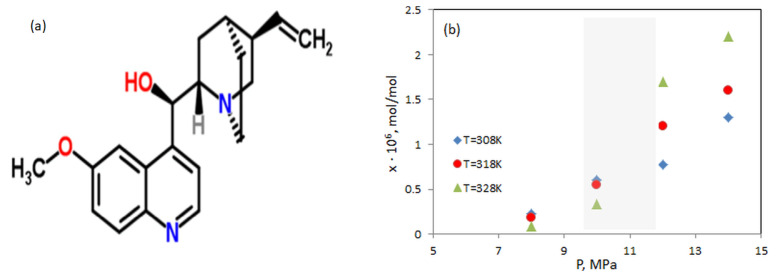
(**a**) Chemical structure of Quinine (19). (**b**) The solubility of quinine in scCO_2_ according to Ref. [5] at different pressures and temperatures. The region of our interest (shadowed) corresponds to the low solubility.

Quinine, anhydrous, 99% (total mass) was bought from ThermoFisher Scientific (produced by Alfa Aesar); CAS 130-95-0. It has a molecular formula of C_20_H_24_N_2_O_2_ and molecular weight 324.4 g/mol. The chemical structure of quinine is shown in Figure 1 which consists of fused aromatic and alkyl rings. Quinine comes in a state of a white crystalline solid and is soluble in alcohol, chloroform and diethyl ether. It is slightly soluble in water and glycerol. The solubility of quinine directly in scCO_2_ was recently studied by Zabihi et al. [5]. They reported that the quinine solubility in scCO_2_ increases with pressure, independent of temperature, since the average intermolecular distance decreases, resulting in stronger interactions between the solute and the solvent molecules. The effect of temperature on solubility is more complex. At lower pressures (8 MPa and 10 MPa), solubility decreases with increasing temperature, however, the inverse trend begins at about 12 MPa and continues to 24 MPa, which was the maximum pressure used in the experiments [5]. In any case, the solubility is of the order of magnitude 10${}^{-6}$ in a molar ratio, which is very small. The solubility for our region of interest in pressure and temperature is shown in Figure 1b according to literature data [5]. In order to increase the quinine solubility, ethanol was selected as a co-solvent.

### 2.2. Instrument

Taylor dispersion technique is based on the diffusive spreading of a small volume of a solution injected into a laminar stream of a carrier fluid. When a carrier solution is pumped through a long capillary tube, the laminar profile for Newtonian fluids has a parabolic velocity distribution. A small volume of a solution is injected into the entrance of a capillary. The flow of the carrier fluid disperses this volume and also induces radial composition gradients which in turn cause radial diffusion. Diffusive fluxes also occur at the front and back sides of the injected volume. These fluxes become important if the radial diffusive fluxes are roughly of the same order of magnitude as the convective axial fluxes. This is the case when either the axial velocity is very low, or when the radial distances are very small. The shape of this distribution at the end of the capillary, known as Taylor peak, is monitored by a detector.

**Figure 2:**
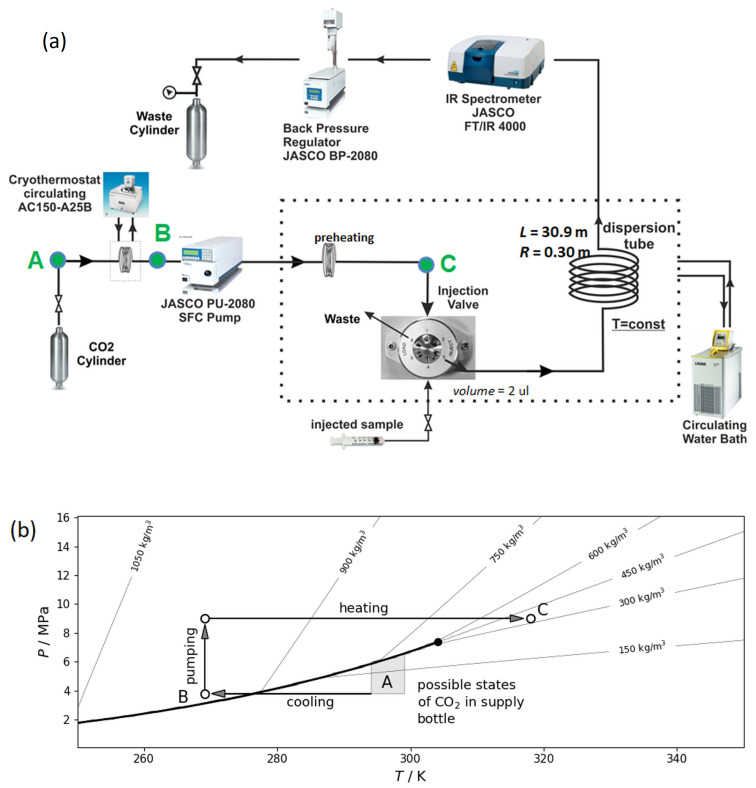
(**a**) Schematic of the high pressure Taylor dispersion apparatus. (**b**) Phase diagram of pure CO_2_ and the representative state points (open circles) in the apparatus. The filled dot is the critical point of CO_2_. The solid curves show the isodensity profiles. Arrows between points A→B→C in both panels outline the experimental procedure.

Figure 2a presents a schematic of the Taylor dispersion instrument used in this and previous studies [6,7,8]. It consists of four modules: a carrier fluid conditioning module, a CO_2_ delivery system with a solute injection valve, an air bath thermostat housing the diffusion capillary and a FT-IR detector.

The first module provided transitions between different states of CO_2_ which are marked in the phase diagram in Figure 2b. CO_2_ was stored in a bottle at a room temperature of and a pressure of about 5.0 MPa in the vapor-liquid equilibrium state (this state is indicated by point A). CO_2_ was drawn from the supply bottle to a cryothermostat (ARCTIC A25B Refrigerated Circulator from Thermo Scientific${}^{\mathrm{TM}}$), cooled to $T_{B}$ = 269.15 K, to condense to the liquid state before it entered the pump (this state is indicated by point B). A low-pulse multi plunger HPLC analytical pump (Jasco PU–2085), working only with liquids, pressurized CO_2_ above its critical pressure $p_{c}$ = 7.38 MPa. Another function of the pump was to push CO_2_ through the dispersion tube with a constant flow rate in the range of 0.12 to 4.0 mL min${}^{-1}$. A heat exchanger with a length of 1.5 m was installed after the pump to heat liquid CO_2_ to its supercritical state before it reached the injection valve (this state is indicated by point C).

Supercritical CO_2_ was delivered to the second module with a constant flow rate through a six-port injection valve (Knauer model D–14163) fitted with a sample loop of volume ΔV into the diffusion capillary. At the beginning of each run, a pulse of solute ΔV = 2 μL was injected into supercritical CO_2_ at the entrance of a long stainless steel dispersion capillary with a circular cross section. The length of the capillary *L* = 30.916 ± 0.001 m was measured with a tape, while its internal radius $R_{0}$ = 0.375 mm was determined by weighing the tube empty and filled with pure water using an analytical balance with a resolution of 0.1 mg.

The capillary was coiled around a grooved aluminum cylinder with a radius $R_{c}$ = 0.175 m. This support provided fixation and temperature stabilization (±0.1 K) due to heat transfer liquid inside the cylinder that was supplied by a second thermostat. Heat exchanger, injection valve and dispersion tube were placed in a box made of 20 mm thick polyurethane foam indicated by a dotted line Figure 2a. A dedicated fan was activated to support temperature homogenization inside the box.

The Taylor peak was monitored at the outlet of the dispersion tube using an FT-IR spectrophotometer (Jasco FT-IR 4100) with a resolution of 4 cm${}^{-1}$ that was equipped with a high pressure demountable cell (Harrick). The FT-IR detector worked at high pressure and the flow was decompressed after the detector. The optical windows of our detector are made of ZnSe supporting a maximum working pressure of 25 MPa. The pressure in the system was controlled by a back pressure regulator (Jasco BP-2080) and measured by pressure sensor JUMO (dTrans p30) with accuracy of ±0.05 MPa.

### 2.3. Selection of Working Wave Numbers

Prior to the experiments, the transmittance of the IR spectra of supercritical CO_2_, ethanol and quinine was investigated. The infrared transmittance spectrum of supercritical CO_2_ was detected with our FT-IR spectrophotometer at *T* = 320 K and *p* = 14 MPa [6]. In the supercritical region, the transmission spectrum of CO_2_ does not change with temperature and pressure. Three regions with the highest transmittance were identified: 800–1200 cm${}^{-1}$, 1400–2100 cm${}^{-1}$ and 2500–3500 cm${}^{-1}$. These are the regions where the presence of other molecules in the flow of sc CO_2_ will be easily detected provided that their absorbance is not negligible. In turn, from the IR spectra of ethanol and ethanol-quinine mixture, wavenumbers were selected where absorbance of IR light is maximal, i.e., transmittance is minimal.

**Figure 3:**
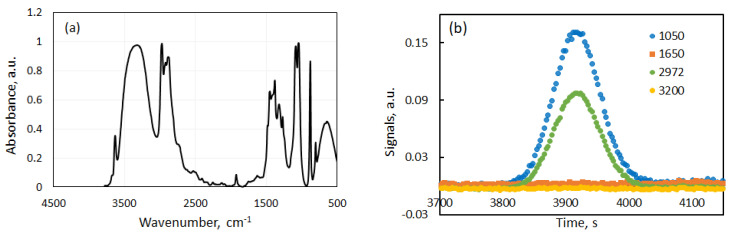
(**a**) Absorbance spectra of ethanol in scCO_2_; (**b**) Taylor peaks observed at different wavenumbers for the mixture containing 0.03% quinine (mass fr) in ethanol injected to scCO_2_ at *p* = 12 MPa and *T* = 313 K.

For the measurement of the absorbance of ethanol, the injection loop was changed from the small volume of 2 μL to the larger one of 2 mL to get a better visual absorbance. With this change of loop, a rough version of the absorbance spectrum shown in Figure 3a was detected. The working wavenumbers are selected in areas where the absorbance of ethanol is maximal and of scCO_2_ is minimal. Figure 3 shows that the regions with high absorbance of ethanol are approximately 1000–1500 cm${}^{-1}$ and 2800–3500 cm${}^{-1}$. After careful analysis and confirmation with literature data [9], the working wavenumbers for ethanol were selected to be 1050 cm${}^{-1}$, 1090 cm${}^{-1}$, 2972 cm${}^{-1}$ and 3331 cm${}^{-1}$.

Quinine is the solid substance and we do not have dedicated facility to measure IR spectrum of crystals. The IR spectrum of several solutions (e.g., quinine hydrochloride) can be found on website [10]. It provides suitable peaks inside the region (1220–1240 cm${}^{-1}$). The contribution of different groups (-OH, -CH, -N-) in the shift of wavenumber in IR spectrum was examined in quinine dissolved in methylene chloride [11]. In this study, the working concentrations of quinine in ethanol are very low, and a large absorbance shift with respect to ethanol was not expected. Using literature data about the wavenumber shift, a preliminary selection of suitable wave numbers was made as 1050 cm${}^{-1}$, 1090 cm${}^{-1}$, 2972 cm${}^{-1}$ and 3200 cm${}^{-1}$. The results of the special tests, shown in Figure 3b, revealed that the absorbtion peak is the largest at the wavenumber 1050 cm${}^{-1},$ while the absorbance observed at 2972 cm${}^{-1}$ and 3200 cm${}^{-1}$ is similar to the baseline. The results below are presented for the working wavenumber 1050 cm${}^{-1}$.

### 2.4. Results Processing

**Figure 4:**
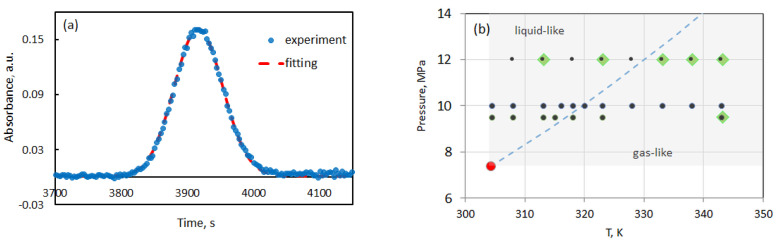
(**a**) Experimental Taylor peak and its fitting when mixture containing 0.03% quinine (mass fr) in ethanol is injected in scCO_2_ at *p* = 12 MPa and *T* = 313 K. (**b**) The pressure-temperature phase diagram on which the dashed curve outlines the Widom line separating the supercritical space in gas-like and liquid-like regions. The green symbols indicate the state points as which diffusion experiments with quinine were conducted in this study while the black dots correspond to the experiments with pure ethanol.

The employed IF-FT detector did not sample the concentration directly, but the absorbance of the solute. As it was discussed previously [7], small changes in concentration are proportional to variations in absorbance. Then, the working equation for the absorption of the solute (in absorption units) averaged over the cross section at the end of the diffusion tube can be written [12] as

(1) $$\begin{array}{ccc} A(t) & = & A_{0}+A_{1}t+A_{2}t^{2}+R\;\left(C\left(t\right)-C_{0}\right)\\ \; & = & A_{0}+A_{1}t+A_{2}t^{2}\\ \; & + & \Delta A\;\sqrt{\frac{t_{R}}{t}}\;\exp\left(-\frac{12\;D\;{\left(t-t_{R}\right)}^{2}}{R_{0}^{2}\;t}\right), \end{array}$$

where the three first terms $A_{0}+A_{1}t+A_{2}t^{2}$ consider the drift and curvature of the baseline due to small concentration and temperature variations; $R={\left(\partial A/\partial C\right)}_{\lambda }$ is the sensitivity of the detector which depends on the wavenumber at which the measurements are conducted; ΔA is the peak height relative to the baseline. In our approach to Taylor dispersion, the diffusion coefficients are obtained by fitting the response curve to the theoretical solution expressed by Equation (1) with subtracting the baseline and offset. The fitting procedure was discussed previously [13]. In the experiments with ethanol and quinine we did not observe an asymmetry of peaks or so-called peak tailing. To illustrate this point, Figure 4a shows a representative peak and its fitting obtained in this work. The state points at which the diffusion coefficients were measured for pure ethanol and ethanol/quinine solutions in scCO_2_ are shown in the pressure-temperature diagram in Figure 4b.

## 3. Results

### 3.1. Diffusion of Ethanol in Supercritical CO_2_

The diffusion coefficients of ethanol in supercritical CO_2_ have been measured previously [14,15] in high density fluids, i.e., on the side of liquid-like state of scCO_2_. Our interest is to examine diffusion coefficients along the isobars crossing the Widom line, i.e., going from liquid-like to gas-like state of scCO_2_ as shown in Figure 4b. Our first results along the *p* = 12 MPa isobar and a detailed description of the experimental challenges were published recently [7]. Since that time, a large number of tests using a fine-tuning of the experimental procedure have been conducted. Here, we present the measurements of the Fick diffusion coefficients along the three isobars: *p* = 9.5 MPa, 10 MPa and 12 MPa, when temperature varies in the range from 304.2 K to 343.2 K. Experimental diffusion coefficient values were averaged over at least five measurements, typically ten samples were taken. The reproducibility of the results was generally good and the relative standard deviation varied from 4% to 9% approaching the Widom line. The measured Fick diffusion coefficient data and corresponding scCO_2_ density are given in Table 1. The density was calculated with the GERG-2008 equation of state, which is the standard reference of the REFPROP 10.0 database [16] maintained by the National Institute of Standards and Technology.

**Figure 5:**
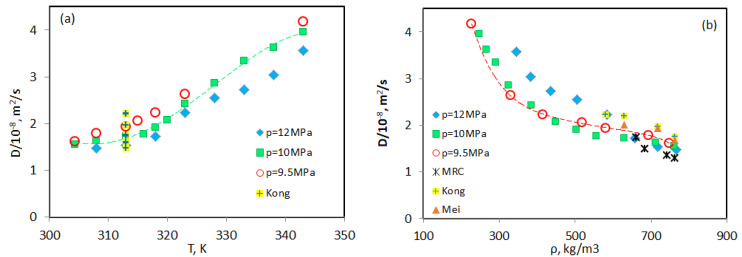
Temperature (**a**) and density (**b**) dependence of Fick diffusion coefficients of ethanol in scCO_2_ along three isobars. The data of Kong et al. [15] and Mei et al. [14] were obtained at various pressures at *T* = 313.2 K. Our results, labeled MRC, are sporadic measurements at *p* = 9, 11, 13 and 14 MPa and *T* = 313.2 K. The dashed lines serve as a guide to the eye.

Figure 5a shows the Fick diffusion coefficient, *D*, of ethanol in scCO_2_ as function of a temperature. Along the studied isobars, all the diffusion coefficients increase with temperature, but in a different manner. In general, *D* increases by more than a factor of two over the considered temperature range. For clarity of presentation, a guide curve to the eye is shown only for *p* = 10 MPa. This curve shows that the Fick diffusion coefficient changes the slope on the temperature dependence at rT∼328 K. It occurs after the crossing the Widom line in the gas-like region of scCO_2_.

With regard to pressure dependence, as a general trend, D decreases with increasing pressure. This can be also seen from the relative location of *D* along different isobars. However, in the critical transition, T∼ 320 K–330 K, (see Figure 5b) this dependence is disturbed, for example, the data of two isobars (*p* = 10 MPa and *p* = 12 MPa) overlap. At a lower temperature, *D* depends only weakly upon the pressure. This can be demonstrated by the measurements by Kong et al. [15] at *T* = 313.2 K, which are also shown in Figure 5a by the symbols with crosses. They illustrate that even with a large amplitude of pressure variation, from 9.5 MPa to 25 MPa, the change in the diffusion coefficient is small. With increasing temperature, the pressure dependence becomes more pronounced.

In the Taylor dispersion experiment, the diffusion coefficient is usually measured as a function of temperature and pressure. The different dependence of fluid properties on pressure and temperature in the supercritical regime complicates data comparison. This is essentially simplified when comparison is made as a function of scCO_2_ density. The density dependence of the diffusion coefficients grouped by pressure is shown in Figure 5b. Figure 5b also includes the measurements by Mei et al. [14] and Kong et al. [15] at *T* = 313.2 K and various pressures; their data fall to the high density region far from the critical transition. We have also added our single results at *T* = 313.2 K and different pressures. In general, these data are lower than those of Kong et al. [15] and Mei et al. [14] but the difference is not significant. In contrast to the temperature dependence, as the density decreases, *D* increases in a similar manner along all the isobars. The increase in *D* is moderate at high densities, approaching the Widom line, the increment changes, and at low densities, *D* grows rapidly. The close inspection of Figure 5b shows that the diffusion curve at the *p* = 9.5 MPa isobar changes slope at ρ∼400 kg/m${}^{3}$ (the green squares). Note that the critical density of scCO_2_ is $\rho_{cr}$ = 421 kg/m${}^{3}$, and this density value is kept along the Widom line. It was shown recently [17] that the small presence of ethanol can visibly shift parameters of the Widom line. One of the first observations of a change in slope or even the appearance of a V-shaped region on a diffusion curve was reported by Nishiumi& Kubota [18], who attributed this to a decrease in thermodynamic factor. Recent molecular dynamic simulations also showed that the thermodynamic factor in mixtures manifests a deep well in the vicinity of the Widom line [8]. At the critical point, the thermodynamic factor is zero per definition.

### 3.2. Diffusion of Quinine with Ethanol as a Co-Solvent in Supercritical CO_2_

It was recently reported that the Fick diffusion coefficient in scCO_2_ may vary strongly at low solute concentrations, for example, in the case of methane [8]. It can be also noted that the content of quinine used in medical and industrial products is very low, for example, tonic water contains slightly less than 0.01% quinine by mass fraction. Here we discuss the diffusion of a small amount of quinine, preliminary dissolved in ethanol, in scCO_2_. As the amount of quinine is small, one can talk about the diffusion of a dilute solution. In this study, the mixture composition is measured as a mass fraction of quinine dissolved in ethanol, expressed as a percentage. This mixture is injected into scCO_2_.

**Figure 6:**
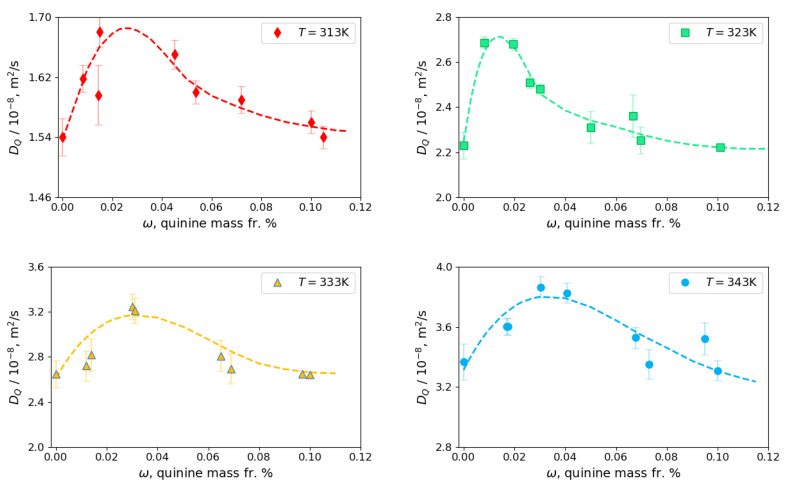
Fick diffusion coefficients of quinine with ethanol as cosolvent in scCO_2_ measured at four different temperatures at *p* = 12 MPa. The dashed curves are the fitting curves $F_{p,T,\;\omega }$. The error bars are standard deviations.

Figure 6 shows the composition dependence of the Fick diffusion coefficients $D_{Q}$ along the isobar *p* = 12 MPa at different temperatures. The symbols indicate the experimental points and the dashed curves, referred to as $F_{p,T,\omega },$ are the fitting curves which are useful for the following discussion. The behavior of the diffusion coefficient at all temperatures displays a similar tendency: it grows at a very low quinine content, reaches a maximum, and then decreases approximately to the value of the diffusion coefficient of pure ethanol in scCO_2_. The comparative analysis of $F_{p,T,\omega }$ curves in different panels demonstrates that the values of the diffusion coefficient $D_{Q}$ grow with temperature. The values of the measured diffusion coefficients $D_{Q}$ are presented in Table 2. The results at *T* = 338 K are excluded from consideration, as they have large scattering. This is attributed to the fact that this point is the closest to the Widom line. The experiments were also carried out at a higher content of quinine, up to 1%; they did not show a significant difference with the diffusion coefficient of pure ethanol or sometimes the measured coefficients were even slightly smaller. Thus, a decrease in the diffusion coefficient at a higher quinine content is evident.

The reasons behind this can be explained by considering different assumptions:

(i) The molecular shape of quinine, shown in Figure 1a, displays a large spatial structure; thus, there is a possibility that the quinine molecules form clusters that tend to be bulky and, in turn, reduce the diffusion coefficient.
(ii) Formation of new compounds with ethanol. Recently was reported [19] that alcohol may attach to quinine which leads to the increase of its molecular weight significantly.
(iii) Higher concentrations of quinine can cause adhesion of its molecules to the wall of the dispersion tube, thus only some of them reach the detector, respectively, a lower diffusion coefficient will be measured.

The last assumption can be discarded, since experiments have shown that at higher quinine content, the Taylor dispersion measures the diffusion coefficient of pure ethanol and not the moderate decrease of the $D_{Q}$ value. These measurements suggest that quinine forms bulky/heavy compounds/clusters that diffuse much more slowly than pure ethanol.

Due to the large amplitude of the diffusion coefficient values, the curves in Figure 6 cannot be accommodated in one figure. In order to understand better the temperature dependence of quinine diffusion, an excess diffusion is examined, that is the difference between $D_{Q}$ and diffusion coefficient of pure ethanol. In fact, fitting curves $F_{p,T,\omega }$ will be used instead of distinct points to better represent and discuss the results. Thus,

(2) $$\Delta D\left(p,T,\omega \right)=D_{Q}\left(p,T,\omega \right)-D_{Q}\left(p,T,\omega =0\right)\approx F_{p,T,\;\omega }-D_{Q}\left(p,T,\omega =0\right).$$

**Figure 7:**
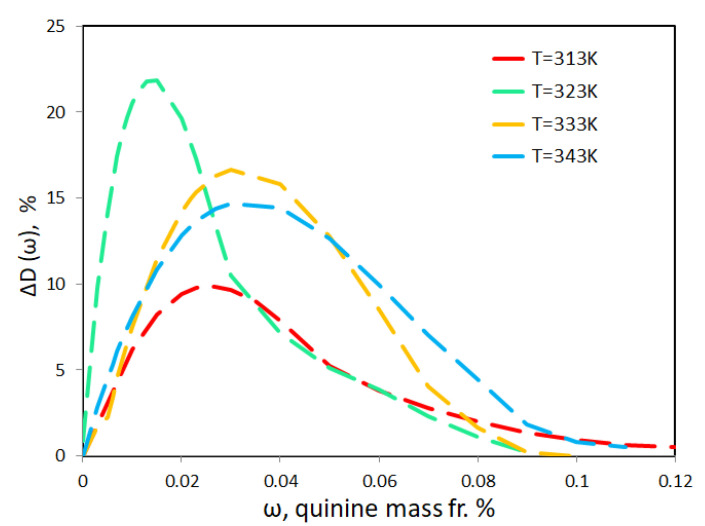
Composition dependence of the excess diffusion coefficient, that is, the difference between $D_{Q}$ and the diffusion coefficient of pure ethanol (see Equation (2)), at different temperatures along the isobar *p* = 12 MPa expressed in percents.

The excess of diffusion coefficient due to presence of quinine, ΔD, is shown in Figure 7 for different temperatures at *p* = 12 MPa. At the lowest measured temperature, *T* = 313 K, the presence of a small content of quinine increases diffusion by 10%. At a higher temperature, *T* = 323 K, the increase in the diffusion coefficient reaches about 25% but rapidly decreases with an increase in the quinine content. Surprisingly, as the temperature rises further, the excess of the diffusion coefficient ΔD decreases. On the one hand, it can be attributed to the crossing the Widom line when scCO_2_ undergoes transition from liquid-like to gas-like state and the density changes sharply. On the other hand, it was also reported by Dawidowicz et al. [19] that exposure to quinine/alcohol mixtures to high temperatures leads to an easier conversion of quinine to new compounds that are structural isomers of quinine. The compounds are hydroxyl derivatives of quinine, which leads to a significant increase in its molecular weight, and the degree of increase depends on the alcohol used. These observations suggest the formation of new complex and more voluminous compounds and, as a consequence, lead to a decrease in the diffusion coefficient.

This effect is more pronounced when the amount of quinine dissolved in ethanol is increased. It is also seen from Figure 7 that at higher temperatures a maximum ΔD(ω) is shifted towards a larger quinine content. It is quite likely that the sharp decrease in the diffusion coefficient shown in Figure 7 is associated with a change in the spatial structure and a subsequent increase in the molecular weight of the sample.

**Figure 8:**
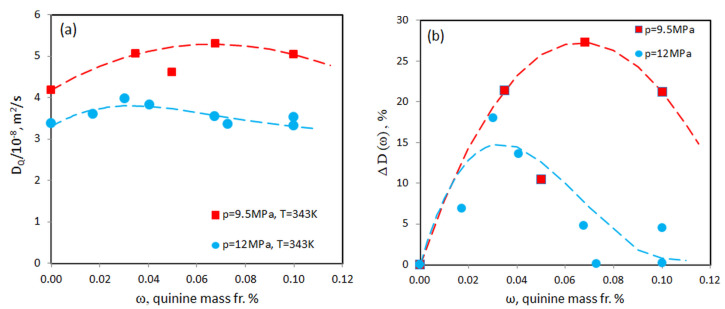
Composition dependence of (**a**) Fick diffusion coefficient and (**b**) excess of the diffusion coefficient of ethanol-dissolved quinine in scCO_2_ along two isobars *p* = 9.5 MPa and 12 MPa at the temperature *T* = 343 K. The dashed curves are the fitting curves $F_{p,T,\;\omega }$.

We also investigated the pressure dependence of the diffusion coefficient. At *T* = 343 K both examined pressures, *p* = 9.5 MPa and *p* = 12 MPa, correspond to the gas-like state of scCO_2_. Figure 8 clearly demonstrates not only a higher value of $D_{Q}$ at a low pressure (panel a), but also a much larger increase in the excess diffusion ΔD due to the presence of quinine (panel b). More detail inspection of panel (b) displays that ΔD at *p* = 9.5 MPa is two times larger than at *p* = 12 MPa. It can be also seen from panel (b) that the maximal point of the excess diffusion ΔD(ω) at the pressure *p* = 9.5 MPa moves to a significantly higher quinine content than at *p* = 12 MPa.

## 4. Conclusions

The diffusion coefficients of quinine in supercritical CO_2_ were measured using a Taylor dispersion technique when quinine is pre-dissolved in ethanol. Our study was focused on the analysis of diffusion coefficients along the isobars crossing the Widom line, which originates from a critical point and divides the supercritical region into liquid-like and gas-like state of scCO_2_.

As a general trend, at either temperature, the diffusion coefficient of quinine $D_{Q}$ first increases noticeably with increasing quinine content in ethanol, reaches a maximum, and then begins to decrease. This occurs over a small range of variation in quinine content. The maximum amount of quinine dissolved in ethanol and used in regular measurements of the diffusion coefficient was ∼0.1%. It is quite likely that a rapid decrease in the diffusion coefficient is associated with a change in the spatial structure, that is, the cluster or compound formation, and a subsequent increase in the molecular weight of the sample.

This research not only promote our expertise in examination of the diffusion process in the supercritical CO_2_ across the Widom line, but also provide the message of using CO_2_ for extraction of quinine with ethanol as co-solvent. The idea to extract quinine from cinchona bark using supercritical CO_2_ was patented in 90th [20] but, for the best our knowledge, it has not been developed. More recent, in 2012, another idea was patented [21] dealing with the extraction of quinine from Peruvian bark by ethanol. The current research highlights a possible combination of the two patented approaches.

## Figures and Tables

**Table 1:** Density (ρ) of scCO_2_, diffusion coefficients (D/10${}^{-8}$m${}^{2}$/s) of ethanol in scCO_2_ and their standard deviations   ($\sigma /10^{-8}$m${}^{2}$/s), measured along three isobars (*p* = 9.5, 10 MPa and 12 MPa) at different temperatures.

T	T	ρ	D	σ	ρ	D	σ	ρ	D	σ
${}^{\circ }$C	K	kg/m${}^{3}$	m${}^{2}$/s	m${}^{2}$/s	kg/m${}^{3}$	m${}^{2}$/s	m${}^{2}$/s	kg/m${}^{3}$	m${}^{2}$/s	m${}^{2}$/s
		*p* = 9.5 MPa			*p* = 10 MPa			*p* = 12 MPa		
31	304	747.3	1.62	0.06	761.0	1.55	0.06			
35	308	694.0	1.79	0.07	712.8	1.64	0.07	767.1	1.47	0.05
40	313	580.0	1.94	0.08	628.6	1.75	0.07	717.8	1.54	0.04
42	315	517.3	2.05	0.08						
43	316				555.6	1.77	0.08			
45	318	414.6	2.23	0.10	502.6	1.97	0.08	657.7	1.73	0.07
47	320				448.28	2.07	2.09			
50	323	329.63	2.64	0.11	384.3	2.43	0.23	584.7	2.23	0.06
55	328				325.07	2.86	0.25	504.51	2.55	0.10
60	333				290.0	3.34	0.28	434.43	2.73	0.08
65	338				265.9	3.62	0.31	382.9	3.14	0.15
70	343	227.2	4.18	0.29	247.77	3.96	0.36	345.9	3.67	0.36

**Table 2:** Diffusion coefficients of ethanol-dissolved quinine in scCO_2_ measured at *p* = 12 MPa at different temperatures. ω is the mass fraction of quinine dissolved in ethanol, expressed as a percentage.

*T* = 313 K		*T* = 323 K		*T* = 333 K		*T* = 343 K	
ω	$D_{q}/10^{-8}$	ω	$D_{Q}/10^{-8}$	ω	$D_{Q}/10^{-8}$	ω	$D_{Q}/10^{-8}$
%	m${}^{2}$/s	%	m${}^{2}$/s	**%**	m${}^{2}$/s	%	m${}^{2}$/s
0.0000	1.470	0.0000	2.231	0.0000	2.648	0.0000	3.368
0.0083	1.548	0.0080	2.687	0.0120	2.720	0.0169	3.602
0.0149	1.610	0.0195	2.680	0.0140	2.820	0.0174	3.602
0.0143	1.526	0.0260	2.510	0.0300	3.249	0.0303	3.864
0.0450	1.580	0.0300	2.480	0.0310	3.210	0.0407	3.826
0.0720	1.520	0.0500	2.310	0.0690	2.696	0.0677	3.529
0.0536	1.530	0.0696	2.254	0.0650	2.810	0.0730	3.350
0.1000	1.490	0.0667	2.360	0.1000	2.650	0.0950	3.520
0.1050	1.470	0.1010	2.221	0.1000	2.640	0.1000	3.308
